# The Effect of Retrieval Focus and Emotional Valence on the Medial Temporal Lobe Activity during Autobiographical Recollection

**DOI:** 10.3389/fnbeh.2013.00109

**Published:** 2013-08-28

**Authors:** Ekaterina Denkova, Sanda Dolcos, Florin Dolcos

**Affiliations:** ^1^Alberta Cognitive Neuroscience Group, University of Alberta, Edmonton, AB, Canada; ^2^Psychology Department, University of Illinois at Urbana-Champaign, Urbana, IL, USA; ^3^Neuroscience Program, University of Illinois at Urbana-Champaign, Urbana, IL, USA; ^4^Beckman Institute for Advanced Science and Technology, University of Illinois at Urbana-Champaign, Urbana, IL, USA

**Keywords:** personal memories, retrieval goal, fMRI, MTL, valence

## Abstract

Laboratory-based episodic memory studies, using micro-events (pictures/words), point to a role of the amygdala (AMY), an emotion-based region, in the encoding and retrieval of emotionally valenced memories. However, autobiographical memory (AM) studies, using real-life personal events, do not conclusively support AMY’s involvement in AM recollection. This could be due to differences in instructions across the AM studies – i.e., whether emotional aspects were explicitly emphasized or not. The present study investigated the effect of retrieval focus on activity in emotion (AMY) and memory (hippocampus – HC) based regions of the medial temporal lobe in 17 subjects, who remembered emotional AMs while event-related fMRI data were recorded. The retrieval focus was manipulated by instructions to focus either on emotional (*Emotion* condition) or on other contextual (*Context* condition) details of the recollected AMs. The effect of retrieval focus according to the valence of AMs was also investigated by involving an equal proportion of positive and negative AMs. There were four main findings, showing both similarities and differences in retrieving positive and negative AMs. Regarding similarities, (1) focusing on Emotion was associated with increased scores of subjective re-experience of emotion and increased activity in the left AMY, for both positive and negative AMs, compared to focusing on Context; (2) the subjective emotional ratings were also positively correlated with bilateral AMY activity for both positive and negative AMs. Regarding differences, (3) focusing on Emotion was associated with increased activity for positive but not for negative AMs in the right AMY, and with (4) opposing patterns of activity linked to the valence of AMs in the left HC – i.e., increased activity for positive and decreased activity for negative AMs. These findings shed light on the role of AMY and HC in emotional AM recollection, linked to the retrieval focus and the valence of memories.

## Introduction

Remembering emotional autobiographical memories (AMs) is an integral part of everyday life that may influence personal well-being and psychological health. Thinking about emotional personal experiences can be used to re-experience positive affect (Bluck and Alea, 2009a) or to reverse negative mood (Josephson et al., 1996; Joormann et al., 2007). However, empirical research has mainly focused on the consequences of reflecting on negative experiences, and has produced contradictory findings. Reflecting on negative events has been found either to reduce the intensity of those experiences (Pennebaker and Graybeal, 2001; Wilson and Gilbert, 2008) or to increase negative affect (Mor and Winquist, 2002; Nolen-Hoeksema et al., 2008; Smith and Alloy, 2009), which may lead to depression. These inconsistent findings might be due, in part, to the type of focus people adopt when recollecting personal experiences, such as focusing on the emotional aspects or on other non-emotional details of the experience (e.g., when and where personal events occurred).

Neuroimaging evidence from “emotional” AM studies^1^(Svoboda et al., 2006) has associated the retrieval of emotional AMs with activity in emotion (amygdala – AMY) and memory (hippocampus – HC) related medial temporal lobe (MTL) regions (Markowitsch et al., 2000; Piefke et al., 2003). Moreover, activity in the AMY and HC was associated with the emotional intensity of memories (e.g., Botzung et al., 2010). This evidence is consistent with findings from laboratory-based episodic memory studies, which have demonstrated greater engagement of both emotion and memory MTL systems during encoding (Dolcos et al., 2004; Kensinger and Corkin, 2004; Kensinger and Schacter, 2006; Sergerie et al., 2006), consolidation (Ritchey et al., 2008), and retrieval (Hamann et al., 1999; Dolcos et al., 2005; Sergerie et al., 2006) of emotional items (reviewed in Dolcos et al., 2012). However, the majority of the “standard” AM studies have not identified AMY involvement during retrieval of personal events or its modulation by emotional intensity (Maguire and Frith, 2003; Addis et al., 2004; but see Daselaar et al., 2008).

The inconsistencies in the AMY engagement during retrieval of AMs could be due to several factors (Greenberg et al., 2005; Denkova et al., 2006; Markowitsch and Staniloiu, 2011). First, it is possible that differences in image acquisition parameters and in statistical analyses (whole-brain vs. ROI analysis) could, at least partially, account for the inconsistencies in AMY activation across these studies (Greenberg et al., 2005). Second, AMY may be involved only in recollections that are sufficiently vivid and strong to elicit a re-experience of the associated emotion (Ochsner and Schacter, 2000). Third, because of the complexity and multifaceted nature of autobiographical events, it is possible that more elaborative processing and cognitive resources needed for their constructive retrieval may attenuate AMY’s involvement during the “standard” AM studies (Denkova et al., 2006; Dolcos et al., 2012). Finally, given that the “standard” AM studies commonly ask participants to retrieve a specific event without a clear and explicit focus on emotional aspects of recollections (Svoboda et al., 2006), and that the episodic laboratory-based memory studies suggest a goal-modulated involvement of the AMY (Smith et al., 2006), it is reasonable to infer that the retrieval instructions given to the participants may influence the AMY engagement during the recollection of personal events. However, this possibility has never been tested and clarified in the AM neuroimaging literature.

The main goal of the present study was to investigate the effect of manipulating the retrieval focus (on emotional vs. on non-emotional, contextual, aspects) on the involvement of emotion (AMY) and memory (HC) related MTL regions during remembering of emotional AMs. In addition, the role of *valence* (positive or negative), which is an important and understudied aspect of AMs, was also investigated. Available evidence suggests that positive and negative AMs may be governed by different mechanisms and lead to different outcomes. Specifically, positive or negative affective biases in AM recollection are closely linked to personal well-being or impaired mental health, respectively. For instance, a positive memory bias in recollections of past personal events and in simulations of future personal events is reported in normal population and healthy aging (Bluck and Alea, 2009b; Denkova et al., 2012; Finnbogadottir and Berntsen, 2012; Szpunar et al., 2012; Rasmussen and Berntsen, 2013), while a negative memory bias is reported in people with/or susceptible to affective disorders, such as depression and post-traumatic stress disorder (PTSD) (MacLeod and Byrne, 1996; Brewin et al., 1999; Nolen-Hoeksema et al., 2008). The valence of AMs can also modulate brain activity. For example, across the few AMs neuroimaging studies considering the valence of memories, recollection of positive personal events has been shown to engage MTL regions linked to greater re-experience of positive events, and orbito-frontal regions, which are involved in the representation of rewarding experiences; on the other hand, recollection of negative AMs engages lateral temporal regions, linked to the processing of negative emotions (Markowitsch et al., 2003; Piefke et al., 2003, but see Vandekerckhove et al., 2005, which failed to observe such an effect).

To investigate these issues, fMRI data were recorded while participants focused either on emotional (*Emotion* condition) or on other contextual (*Context* condition) details during elaboration of recollected positive and negative AMs. In the Emotion condition, participants were instructed to remember past events by focusing on the emotional aspects of their recollections, whereas in the Context condition participants were instructed to remember past events by focusing on other, non-emotional, contextual details (e.g., details about the time and place of personal events). Based on the extant evidence, we made the following predictions. Concerning the behavioral results, we predicted increased emotional ratings for both positive and negative AMs when focusing on emotional details of the recollected AMs. Concerning the fMRI results, we predicted both similar and dissociable effects in the MTL regions, linked to the retrieval focus and the emotional valence of the AMs. Specifically, we expected overall similar greater engagement of MTL activity during AMs recollection in the *Emotion* condition than in the *Context* condition, for both positive and negative memories. We also expected a link between increased emotional ratings and AMY activity in the *Emotion* condition. Finally, based on evidence that positive and negative AMs may be governed by different mechanisms, we also expected a dissociable engagement of MTL regions according to the valence of memories, possibly with positive memories leading to greater MTL engagement, particularly in the *Emotion* condition.

## Materials and Methods

### Participants

Eighteen right-handed native English speaking young adults with no history of neurological, psychological, or psychiatric illness participated in this study (six men; age range 18–46, mean = 26 years, SD = 7.02). One subject dropped out the study after the first run of the fMRI session, hence, data from 17 subjects (six men, mean age = 26.06 years; SD = 7.20) were analyzed. The experimental protocol was approved by the Institutional Health Research Ethics Board, and all participants provided written informed consent and received payment for their participation.

### Collection and selection of emotional autobiographical memories

Personal memories were elicited from each participant during an interview performed ∼5 weeks prior to the fMRI session, similar to other AM neuroimaging studies (Markowitsch et al., 2000; Maguire and Frith, 2003; Piefke et al., 2003; Addis et al., 2004; Botzung et al., 2008). This procedure allows increased control over the properties of the memories to be used in different trial types, as compared to involving AM retrieval directly in the scanning session (Maguire, 2001; Cabeza and St Jacques, 2007; St Jacques, 2012). In addition, it attenuates the disadvantage of reactivation by interposing sufficient time between the pre-scan interview and the subsequent scanning session (Maguire and Mummery, 1999). We used an autobiographical memory questionnaire (AMQ) specifically constructed to target the assessment of emotional personal episodes and their recollective properties (Denkova et al., 2012). The AMQ comprised a list of 115 verbal cues for distinct life events (e.g., “the birth of a family member,” “being hospitalized”), resulted from a combination and extension of lists employed by other authors (Levine et al., 2002; Markowitsch et al., 2003; Sharot et al., 2007). For each cue, participants were asked to remember a unique episode from their life, that occurred in a specific place and time (e.g., one instance when s/he played in a specific basketball game), rather than remembering general or repeated events (e.g., playing basketball in high school). Importantly, the memories had to be accompanied by the recollection of being personally involved, rather than hearing about them from others. Upon recollection, participants were asked to provide a brief description of the memory, which was then used as a personalized memory cue during the fMRI scanning; at the time of collecting the AMs, participants were naïve to the specific purpose of the pre-scanning interview. To assess phenomenological characteristics of each event, participants dated the memory and rated it on several Likert scales, similar to those used in other AM studies (Addis et al., 2004; Greenberg et al., 2005), as follows: Emotional Valence (using a 7-point scale: −3 = very negative, 0 = neutral, and +3 = very positive), Emotional Intensity, Personal Significance, Vividness (i.e., the amount of visuo-perceptual details), the amount of Contextual Details, and the Frequency of Retrieval (all of the latter used a 7-point scale: 1 = not at all, 7 = extremely).

For each participant, we selected the 40 most emotional memories (20 positive and 20 negative), based on the ratings provided on the AMQ (i.e., rated 2 or 3 and −2 or −3, respectively). Half of the selected memories, with an equal proportion of positive and negative AMs, were assigned to an *Emotion* Retrieval Focus AM condition (10 positive and 10 negative), and the other half of AMs were assigned to a *Context* Retrieval Focus AM condition (10 positive and 10 negative). This resulted in four AM event types: Emotion Focus Positive, Emotion Focus Negative, Context Focus Positive, and Context Focus Negative Memories. To ensure that any differences between the two retrieval foci/goals during the fMRI session would not be due to initial differences in the properties of the memories assigned to the Emotion and Context conditions, the positive and negative memories of the two conditions were matched as closely as possible in terms of phenomenological properties (see Table 1). The descriptions provided by the participants were used to create memory cues specific for each participant. If necessary, the descriptions were slightly adapted to be matched as closely as possible in terms of length and grammatical complexity. Four other memories were also selected and used in practice trials before the fMRI session.

**Table 1:** **Phenomenological properties of the selected autobiographical memories**.

	Negative		*t* test, *p*	Positive		*t* test, *p*
	Emotion	Context		Emotion	Context	
Emotional intensity	5.68 (0.51)	5.70 (0.52)	0.60	5.25 (0.83)	5.29 (0.82)	0.20
Vividness	5.19 (0.80)	5.15 (0.67)	0.47	5.54 (0.61)	5.49 (0.73)	0.52
Contextual details	4.94 (1.06)	4.92 (1.12)	0.72	5.38 (1.17)	5.30 (1.25)	0.32
Personal significance	4.42 (1.02)	4.32 (1.08)	0.09	4.69 (1.01)	4.73 (0.93)	0.61
Frequency of rehearsal	3.33 (1.04)	3.32 (0.96)	0.87	3.64 (0.96)	3.54 (0.95)	0.20

*Standard deviations are given in parentheses. There were no significant differences between memories assigned to the Emotion condition and those assigned to the Context condition*.

### fMRI tasks

The fMRI session comprised two AM tasks, according to retrieval focus (*Emotion* and *Context*), and a semantic memory (SM) control task. Given the goal of the present investigation, in each AM task, half of the memories were positive and the other half were negative. Just before performing the fMRI tasks, participants were given detailed instructions and examples for the tasks they had to perform in the scanner. In addition, participants performed practice trials in order to familiarize themselves with the tasks and to ensure that they understood the instructions.

#### The autobiographical memory tasks

Participants were asked to retrieve the memories associated with each personalized memory cue by either focusing on emotional (*Emotion* condition) or focusing on other contextual (*Context* condition) aspects of their positive and negative memories. For the *Emotion* condition, participants were instructed to remember the specific event and focus on the emotional aspects of their memories, including associated sensations and feelings that they may have triggered (e.g., butterflies in the stomach, palpitations). For the *Context* condition, participants were instructed to remember the specific event and focus on other, non-emotional, contextual aspects of their memories, by retrieving as many contextual details as possible (e.g., about where and when the event occurred, who else was involved, etc.). Each memory cue was preceded by an instruction cue, as follows: “Remember Emotion,” for the Emotion condition, and “Remember Context,” for the Context condition, respectively. Once the memory cue appeared on the screen, participants had to indicate by a button press that they recognized the cue as belonging to them, and then continued remembering details of the event until cued to rate the recollected memory. Each recollection was rated on three five-point Likert scales including Emotional Intensity, Vividness, and Reliving (1 = very low; 5 = very high). The participants were instructed to make quick (spontaneous) and accurate responses and to use the whole scale.

#### The semantic memory control task

In line with other AM functional neuroimaging studies (Greenberg et al., 2005; Young et al., 2012), we used a control condition involving SM retrieval. Specifically, the SM task involved generation of exemplars from 20 different semantic categories (e.g., musical instruments, sports, vegetables) (Battig and Montague, 1969), which like the AM retrieval involves search in memory and extended retrieval time. The participants were presented with a semantic category name cue (e.g., fruits, vegetables) and instructed to recall as many exemplars as possible for each category. Each semantic category cue was preceded by an instruction cue (“Generate Examples”). Once the category cue appeared on the screen, participants had to indicate by a button press that they started recalling exemplars from the category, and then they continued recalling until cued again for memory ratings. To be consistent with AM conditions, each exemplar generation was rated on three five-point Likert scales appropriate for SM generation – i.e., Vividness, Difficulty of the task (1 = very low; 5 = very high), and approximate Number of the recalled items (1 = 1 to 3 items; 5 = 15 or more items).

### fMRI design and procedure

**Figure 1:**
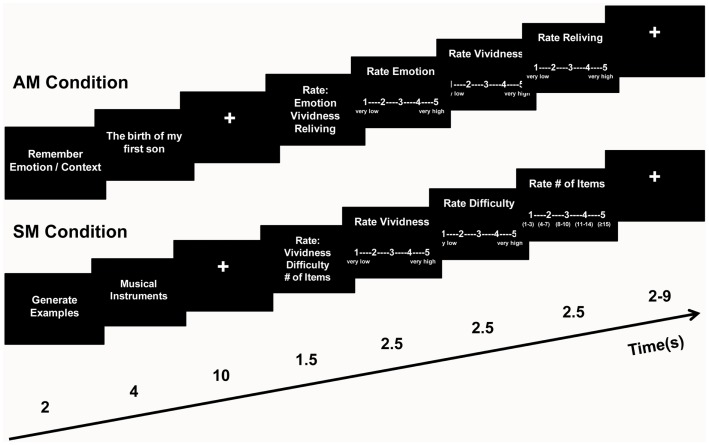
**Diagram of the task**. During the Autobiographical Memory (AM) conditions, participants remembered highly emotional personal memories by focusing either on emotional (Emotion) or on other contextual (Context) aspects of their recollections, and then rated each AM for Emotional Intensity, Vividness, and Reliving on five-point scales. During the control Semantic Memory (SM) condition, participants generated as many exemplars as possible from a given semantic category, and then rated each of them for Vividness, Difficulty, and Number of Items on five-point scales.

The AM conditions and the SM control condition had the same general structure (see Figure 1). Each trial began with an instruction screen for 2 s, immediately followed by a memory cue for 4 s. After the cue offset, a fixation screen was presented for 10 s during which participants elaborated their personal memories or generated exemplars. The end of the retrieval period was marked by the presentation of an instructions screen for upcoming ratings, for 1.5 s. Then, each of the three ratings was presented for 2.5 s and in a counterbalanced order across trials. The ratings were followed by an inter-trial interval of variable duration (2–9 s, average = 6 s), before the beginning of the next trial.

The scanning session was divided into two parts of four runs. Each run started with a 6-s fixation, to allow stabilization of the fMRI signal, and comprised five trials from each condition (Emotion, Context, and Semantic). To avoid induction of longer-lasting effects, the trials within each run were pseudo-randomized, so that no more than two consecutive trials of the same type were presented. To prevent possible biases resulted from using the same run order, participants were assigned different run orders. Similar to other AM neuroimaging studies (Greenberg et al., 2005), in order to increase statistical power, the four runs from the first part were immediately repeated in the second part of the scanning session, and the order of the runs was counterbalanced across participants. Stimuli were projected on a screen directly behind the subjects’ heads within the scanner, which they viewed through a mirror.

All stimuli appeared in white letters against a black background created in Adobe Photoshop. The CIGAL software (http://www.nitrc.org/projects/cigal/) was used for stimulus presentation and collection of behavioral responses during the fMRI session. All responses were made on a four-button MRI-compatible response box placed under the subject’s right hand; the fifth rating was indicated by the participants with a double click on button #1.

### MRI data collection

MRI data were recorded using a 1.5-T Siemens Sonata scanner. The anatomical images were 3D MPRAGE anatomical series (repetition time [TR] = 1600 ms, echo time [TE] = 3.82 ms, field of view [FOV] = 256 mm × 256 mm, number of slices = 112, voxel size = 1 mm × 1 mm × 1 mm). The functional images consisted of series of images acquired axially using an echoplanar sequence (TR = 2000 ms, TE = 40 ms, FOV = 256 mm × 256 mm, number of slices = 28, voxel size = 4 mm × 4 mm × 4 mm).

### Behavioral data analysis

To investigate the effect of retrieval focus on the qualities of the negative and positive remembered memories, we performed repeated-measures ANOVA with three factors: Focus (Emotion, Context), Valence (Negative, Positive), and Ratings (Emotional Intensity, Reliving, Vividness).

### fMRI data analysis

Statistical analyses, performed with SPM2 (Statistical Parametric Mapping), were preceded by the following pre-processing steps: Quality Assurance, TR Alignment, Motion Correction, Coregistration, Normalization, and Smoothing (8 mm full-width half maximum isotropic Kernel). At the individual level, each event was modeled by the canonical hemodynamic response function (*hrf*) and its temporal derivate. Movement parameters calculated during the realignment were included as parameters of no interest to control for movement artifacts. According to previous AM neuroimaging studies (Addis et al., 2007), to allow for reading the cue, the *hrf* was time-locked to 2 s (1TR) following the onset of the memory cues, in the Emotion and Context AM conditions, and 1 s (0.5TR) after the onset of the category cue, in the SM condition. Consistent with these, investigation of the RT data showed that the recognition of the AM cues occurred at an average RT of 1.67 s (±0.44), and the beginning of exemplar generation in the SM condition occurred at an average RT of 1.03 s (±0.40). For the fMRI analysis according to focus and valence, we selected randomly only 10 SM events to match the numbers of each of the four AM event types. Individual contrasts were computed directly between the different AM event types (e.g., Emotion Positive vs. Context Positive, Emotion Negative vs. Context Negative). These individual contrasts were then entered into group-level *t* tests, to perform random-effects analyses. The SPM analyses were complemented by analyses performed with in-house MATLAB tools (Dolcos and McCarthy, 2006; Denkova et al., 2010), which allowed extraction of the fMRI signal and examination of the time course of activity related to different conditions, across the whole length of the trials.

To investigate the effects of retrieval focus on positive memories in MTL regions, we compared positive AMs with Emotion focus and positive AMs with Context focus (Emotion Positive > Context Positive and Context Positive > Emotion Positive). Similarly, to investigate the effects of retrieval focus on negative memories, we performed the following comparisons: Emotion Negative > Context Negative and Context Negative > Emotion Negative. Additionally, to also investigate the effect of valence within each retrieval focus, we compared positive and negative AMs with Emotion focus (Emotion Positive > Emotion Negative and Emotion Negative > Emotion Positive) and positive and negative AMs with Context focus (Context Positive > Context Negative and Context Negative > Context Positive).

The common effects of the retrieval focus on both positive and negative memories were investigated through conjunction analyses [e.g. (Emotion Positive vs. Context Positive) ∩ (Emotion Negative vs. Context Negative)]. The dissociating effects of the retrieval focus and valence were investigated through interaction analyses using paired *t* tests [e.g., (Emotion Positive vs. Context Positive) vs. (Emotion Negative vs. Context Negative)], whose outputs were inclusively masked with the corresponding direct effect (e.g., Emotion Positive vs. Context Positive) to ensure that the interaction difference is due to an existing increased difference in the comparisons/contrasts of interest. Finally, to investigate whether differential activity in the MTL according to the focus of retrieval is linked to differences in the subjective feeling of emotion, linear regression analyses were performed between difference scores in self-reported emotion ratings (Emotion ratings minus Context ratings) and MTL activity for Emotion > Context contrasts, for positive and negative AMs, respectively.

As the main goal of the study was to investigate the effects of retrieval focus and valence of AMs on emotion- and memory-related MTL regions, we used anatomical masks of the AMY and HC, based on the Wake Forest University Pick Atlas toolbox. Overall, for all ROI analyses in the AMY and HC, identified as regions of *a priori* interest, we used a statistical threshold of *p* < 0.05 and an extent threshold of five contiguous voxels. For completeness, we also report results of whole-brain analyses for regions outside of the MTL. For these analyses, an intensity threshold of *p* < 0.001 was used for the specific direct contrasts and a threshold of *p* < 0.005 was used for the interactions; the extent threshold was of 10 contiguous voxels.

## Results

### Behavioral results

#### Increased re-experiencing of emotion for both positive and negative AMs in the Emotion condition

**Figure 2:**
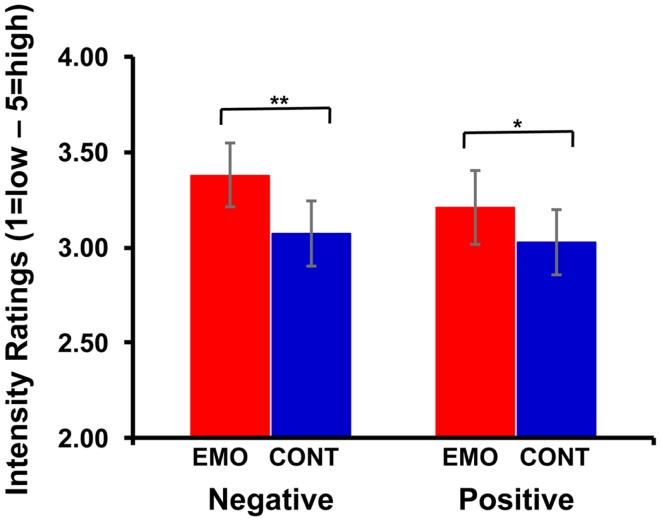
**Increased subjective re-experiencing of emotion during Emotion focus retrieval**. Self-reported ratings for emotional intensity were higher in the Emotion (EMO) than in the Context (CONT) condition, for both negative (**p < 0.005) and positive (*p < 0.05) autobiographical memories.

Repeated-measures ANOVA revealed a main effect of focus [*F*_(1,16)_ = 6.33, *p* = 0.02], indicating higher ratings for the memories retrieved with the Emotion focus. This effect was qualified by a focus x ratings interaction [*F*_(1, 16)_ = 4.12, *p* = 0.03], driven by an increase only for the emotional intensity ratings of AMs retrieved with an emotional focus (Emotion condition); no significant increase was observed in the Reliving and Vividness ratings. The increase was significant for both positive (3.21 vs. 3.03, *p* = 0.02) and negative (3.38 vs. 3.07, *p* = 0.003) AMs (see Figure 2), thus precluding a significant focus x valence x ratings interaction [*F*_(1, 16)_ = 1.30, *p* = 0.29]. Overall, the ratings assessed immediately after recollecting AMs during the scanning sessions showed that the manipulation of the retrieval focus (Emotion vs. Context) dissociated the subjective re-experience of emotion for both positive and negative memories, without affecting significantly the subjectively reported ratings for Reliving and Vividness.

### fMRI results

The present fMRI results revealed both common and dissociable effects of retrieval focus (Emotion vs. Context), linked to the valence of AMs, in emotion (AMY) and memory (HC) related MTL regions.

### Common effects of retrieval focus on positive and negative AM retrieval in AMY

#### Increased activity in left AMY for both positive and negative memories in the Emotion condition

**Figure 3:**
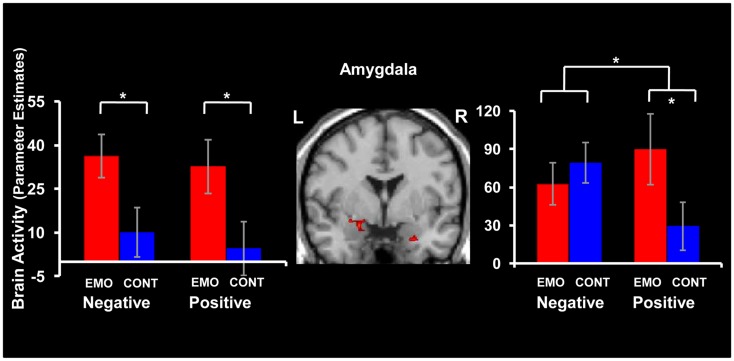
**Common and dissociable effects of retrieval focus in the amygdala (AMY), for positive and negative memories**. Focusing on Emotion (EMO) compared to focusing on Context (CONT) led to similar increases of activity in the left AMY (left panel) for both positive and negative memories; dissociable patterns of activity linked to valence were observed in the right AMY (right panel). The conjunction and interaction maps for the left and right AMY, respectively, are superimposed on a high resolution brain image displayed in a coronal view. The bar graphs represent the contrasts estimates extracted from representative voxels in the left and right AMY, respectively. The error bars correspond to the standard errors of the means. L = Left, R = Right.

Manipulation of the retrieval focus was associated with increased activity in the left AMY for both positive and negative memories, in the Emotion compared to the Context condition (Emotion > Context) (see Figure 3 and Table 2). Common engagement of this left AMY area during retrieval of both positive and negative AMs was revealed by the following conjunction analysis: [(Emotion Positive > Context Positive) ∩ (Emotion Negative > Context Negative)].

**Table 2:** **Activations in MTL ROIs linked to the retrieval focus and emotional valence of memories**.

MTL regions	Side	Talairach coordinates			*T* score	Cluster size
		*x*	*y*	*Z*		
**A. DIRECT CONTRASTS**						
Emotion Negative vs. Context Negative
Amygdala	L	−20	−4	−10	2.76	13
	L	−32	−1	−10	2.43	
Emotion Positive vs. Context Positive
Amygdala	L	−32	−1	−10	3.56	32
	R	20	3	−24	3.57	35
Hippocampus	L	−32	−16	−16	3.99	67
	R	32	−27	−5	3.24	52
Context Negative vs. Emotion Negative
Hippocampus	L	−32	−39	−1	3.56	8
	L	−40	−16	−20	2.32	8
Emotion Positive vs. Emotion Negative
Hippocampus	L	−36	−24	−9	3.39	21
Context Negative vs. Context Positive
Amygdala	L	−32	−8	−13	3.59	5
	R	24	−1	−20	3.33	35
Hippocampus	L	−32	−12	−9	4.33	67
	R	32	−12	−13	4.47	59
**B. CONJUNCTION**						
(Emotion Negative vs. Context Negative) ∩ (Emotion Positive vs. Context Positive)
Amygdala	L	−32	−1	−10	3.56	12
	L	−24	−1	−13	2.79	
**C. INTERACTIONS**						
(Emotion Positive vs. Context Positive) vs. (Emotion Negative vs. Context Negative)
Amygdala	R	24	3	−24	2.66	11
Hippocampus	L	−32	−16	−16	5.06	41
Hippocampus	R	32	−31	−5	2.97	24
(Context Negative vs. Emotion Negative) vs. (Context Positive vs. Emotion Positive)
Hippocampus	L	−36	−16	−16	3.60	7
	L	−32	−27	−5	2.70	8
(Emotion Positive vs. Emotion Negative) vs. (Context Positive vs. Context Negative)
Hippocampus	L	−32	−16	−16	5.06	19
(Context Negative vs. Context Positive) vs. (Emotion Negative vs. Emotion Positive)
Amygdala	R	24	−1	−23	2.61	8
Hippocampus	L	−32	−16	−16	5.06	46
Hippocampus	R	32	−32	−9	3.20	32

*Significant activations resulting from direct contrasts, conjunctions, and interactions analyses in a priori targeted MTL ROIs (AMY and HC) are reported. An intensity threshold of p < 0.05 and an extent threshold of 5 contiguous voxels were used for all ROI analyses. For the conjunction analyses, a threshold of p < 0.05 was used in each of the contributing contrast, and for the interaction analyses the interaction contrast was inclusively masked with the corresponding direct contrast set up at p < 0.05 (see Materials and Methods for details). MTL = Medial Temporal Lobe; L = left, R = right*.

#### AMY activity linked to subjective re-experiencing of emotion for both positive and negative memories

**Figure 4:**
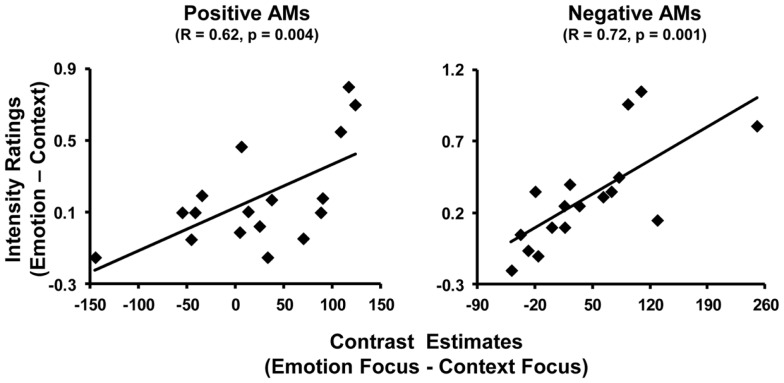
**Positive correlation between activity in the AMY and self-reported emotional ratings**. Differential activity in the AMY for Emotion and Context focuses was positively related to the difference in emotion intensity ratings between Emotion and Context conditions during recollection of positive (left panel) and negative (right panel) memories. The scatter plots are based on contrast estimates for Emotion – Context conditions extracted from the peak voxel of the areas showing the co-variation with the differences in ratings, in the left AMY. Similar co-variations were also identified in right AMY (not shown).

The difference in AMY activity between Emotion and Context (Emotion > Context) was positively correlated with the difference in emotional intensity ratings between Emotion and Context (intensity ratings in Emotion condition minus intensity ratings in Context condition) (see Figure 4). This effect was observed in both left and right AMY and for both positive and negative AMs. For negative memories, the AMY areas showing the correlation with the ratings also overlapped with the area showing greater activity in the Emotion than in the Context condition, in the left (*x* = −28, *y* = −1, *z* = −10; *R* = 0.72, *p* = 0.001), but not in the right (*x* = 24, *y* = −1, *z* = −13; *R* = 0.53, *p* = 0.03) hemisphere. For positive memories, the areas of left and right AMY showing the correlations with emotional ratings (*x* = −28, *y* = 7, *z* = −17; *R* = 0.62, *p* = 0.004, and *x* = 24, *y* = 7, *z* = −21; *R* = 0.62, *p* = 0.004, respectively) showed only very little overlaps with the areas showing increased activity for Emotion compared to the Context condition.

### Dissociable effects of retrieval focus on positive and negative AM retrieval in AMY and HC

#### Increased right AMY activity for positive but not for negative memories in the Emotion condition

Focusing on Emotion compared to Context led to a dissociable pattern of activity in the right AMY for positive and negative AMs (see Figure 3 and Table 2). Specifically, retrieval of positive memories was associated with greater activity in the Emotion than in the Context condition (Emotion Positive > Context Positive), while retrieval of negative memories produced similar effects in the Emotion and Context conditions (Emotion Negative = Context Negative). These effects were confirmed by a repeated-measures ANOVA, performed on the extracted signal, which revealed a significant valence x focus interaction [*F*_(1, 16)_ = 6.84, *p* = 0.02]. This interaction was driven by a significant increase in the Emotion condition compared to the Context condition for positive (*p* = 0.01) but not for negative (*p* = 0.44) AMs.

#### Opposing patterns of activity in the left hippocampus for positive and negative memories

**Figure 5:**
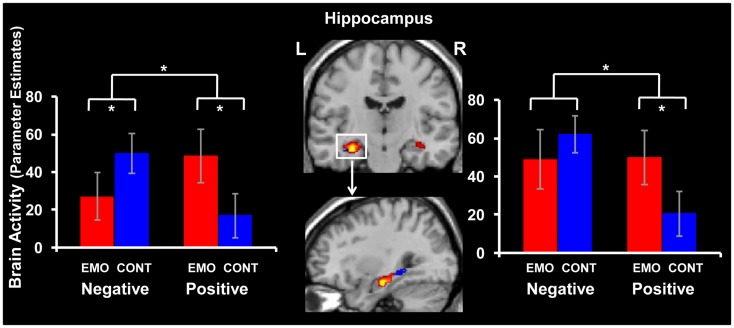
**Dissociable effects of retrieval focus in the hippocampus (HC) for positive and negative memories**. Focusing on Emotion (EMO) compared to focusing on Context (CONT) led to increased activity for positive and decreased activity for negative memories in the left hippocampus (HC) (left panel). Although, overall, a similar effect was observed in the right HC (right panel), only the increased activity for positive memories was statistically significant. The interaction map for the left and right HC is superimposed on a high resolution brain image displayed in a coronal view (top panel). The sagittal view (bottom panel) illustrates the posterior extension of activity in the left HC (blue blob), for negative memories (Context Negative > Emotion Negative). The bar graphs represent the contrasts estimates extracted from representative voxels of the interaction effects in left and right HC. The error bars correspond to the standard errors of the means. L = Left, R = Right.

Comparison of the effect of retrieval focus also identified increased activity for positive (Emotion Positive > Context Positive) and decreased activity for negative (Emotion Negative < Context Negative) AMs in the left HC, in the Emotion condition (see Figure 5 and Table 2). The decreased activity observed for the negative AMs in the left HC extended more posteriorly to the parahippocampal gyrus. Although similar overall patterns of activity were observed in the right HC, only the increased response for positive AMs when focusing on Emotion compared to Context was significant (Emotion Positive > Context Positive).

These effects were confirmed by repeated-measures ANOVAs performed on the extracted signal from peak voxels, which, in the left HC, revealed a significant valence x focus interaction [*F*_(1, 16)_ = 12.94, *p* = 0.002]. This interaction was driven by a significant increase for positive memories (*p* = 0.009) and a significant decrease for negative memories (*p* = 0.048) in the Emotion compared to the Context condition. Similarly, the effect in the right HC was confirmed by a repeated-measures ANOVA revealing a significant valence x focus interaction [*F*_(1, 16)_ = 6.66, *p* = 0.02], which was driven by a significant increase for positive memories (*p* = 0.03) in the Emotion compared to the Context condition.

### Effects of retrieval focus on brain regions outside of the MTL

While the primary focus in the present report concerns the effects of retrieval focus on the MTL activity during retrieving positive and negative AMs, for completeness, results of whole-brain analyses are also reported (see Table 3).

**Table 3:** **Whole-brain activations linked to retrieval focus and valence of memories**.

Brain regions	BA	Side	Talairach coordinates			*T* score	Cluster size
			*x*	*y*	*z*		
**A. DIRECT CONTRASTS**							
Emotion Positive vs. Context Positive
Inferior frontal gyrus	47	R	28	35	−5	6.08	54
Anterior cingulate	10	R	20	47	1	5.37	
Middle temporal gyrus	21	L	−56	0	−7	5.63	26
Superior temporal gyrus	38	L	−56	11	−7	4.83	
Precuneus	7	L	−4	−59	62	4.97	10
Inferior frontal gyrus	44	L	−55	12	10	4.91	10
Inferior frontal gyrus	47	L	−36	19	−8	4.85	41
Emotion Positive vs. Emotion Negative
Precentral gyrus	4	L	−44	−18	34	4.68	11
Context Negative vs. Context Positive
Inferior frontal gyrus	47	L	−36	19	−14	4.84	13
Hippocampus		L	−32	−12	−9	4.40	5
Hippocampus		R	32	−12	−13	4.47	5
**B. INTERACTIONS**							
(Emotion Positive vs. Context Positive) vs. (Emotion Negative vs. Context Negative)							
Middle temporal gyrus	21	L	−60	−4	−3	6.20	13
Hippocampus		L	−32	−16	−16	5.06	17
Caudate (tail)		L	−24	−42	13	4.98	24
Postcentral gyrus	3	L	−48	−18	34	4.51	28
Middle frontal gyrus	10	R	44	42	−5	4.40	14
(Context Negative vs. Emotion Negative) vs. (Context Positive vs. Emotion Positive)							
Caudate (tail)		L	−24	−42	13	4.98	13
Hippocampus		L	−36	−16	−16	3.60	7
(Emotion Positive vs. Emotion Negative) vs. (Context Positive vs. Context Negative):							
Hippocampus		L	−32	−16	−16	5.06	10
Cingulate gyrus	31	L	−24	−46	13	4.64	17
Postcentral gyrus	3	L	−48	−18	34	4.51	28
(Context Negative vs. Context Positive) vs. (Emotion Negative vs. Emotion Positive):							
Hippocampus		L	−32	−16	−16	5.06	19
Caudate (tail)		L	−24	−42	13	4.98	13
Postcentral gyrus	3	L	−48	−18	34	4.51	15
Middle frontal gyrus	10	R	40	42	−5	4.32	11

*Significant activations resulting from whole-brain analyses investigating direct contrasts and interactions are reported. For direct contrasts, a threshold of p < 0.001 was used, and for interactions a threshold of p < 0.005 was used. For the interaction analyses, the interaction contrast was inclusively masked with the corresponding direct contrast set up at p < 0.05. A cluster size of 10 contiguous voxels was used in all analyses, except for the MTL, where a cluster size of 5 contiguous voxels was used. BA = Brodmann’s area; L = left, R = right*.

## Discussion

The present study investigated the behavioral and brain imaging effects of retrieval instructions, linked to the valence of memories, on AM recollection. There were four main findings, showing both similarities and differences in retrieving positive and negative AMs. Regarding similarities, (1) the behavioral data showed that focusing on Emotion was associated with increased scores of subjective re-experience of emotion, and the fMRI data identified increased activity in the left AMY, for both positive and negative AMs, compared to focusing on the Context; (2) the subjective emotional ratings were also positively correlated with bilateral AMY activity for both positive and negative AMs. Regarding differences, (3) focusing on Emotion was associated with increased activity for positive but not for negative AMs in the right AMY, and with (4) opposing patterns of activity linked to the valence of AMs in the left HC – i.e., increased activity for positive AMs and decreased activity for negative AMs. These findings will be discussed in turn below.

### Common effects of retrieval focus on positive and negative AM retrieval in AMY

(1) Manipulation of the retrieval focus was associated with increased activity in the left AMY for both positive and negative memories in the Emotion compared to the Context condition. This finding is consistent with the emotion research suggesting that the AMY’s engagement can be modulated by attention, current goals, and task demands (Blair et al., 2007; Lieberman et al., 2007; Shafer et al., 2012). Moreover, this finding extends the available evidence by revealing that this effect also applies to the retrieval of positive and negative AMs. This is important because the evidence that left AMY activity is susceptible to and acts in accordance with the current retrieval goals clarifies inconsistent findings regarding its involvement in previous AM studies. Typically, these studies emphasize the effortful reconstruction of personal episodes that occurred at a specific time and place (Maguire, 2001; Svoboda et al., 2006), and do not systematically or explicitly probe the emotionality of the recollected memories.

(2) The AMY activity was also positively correlated with the emotional intensity ratings in the Emotion vs. Context condition, so that greater engagement of the AMY when focusing on emotional compared to the contextual details was associated with greater subjective re-experience of emotion of the recollected AMs. This finding provides, therefore, a direct link between AMY activity and subjective affective re-experience, which was not observed in previous “standard” AM studies (Maguire and Frith, 2003; Addis et al., 2004) probably due to the absence of a clear emotional focus during retrieval.

### Dissociable effects of retrieval focus on positive and negative AM retrieval in AMY and HC

(3) Retrieval of positive AMs was associated with increased right AMY activity in the Emotion compared to the Context condition, while retrieval of negative AMs produced similar effects in the right AMY. The latter finding suggests that the right AMY activity is not modulated by the current retrieval goals in the case of negative AMs. This is in contrast to the left AMY activity, which is sensitive to the current retrieval goals in the case of both positive and negative AMs, and altogether these findings suggest a hemispheric dissociation in the AMY regarding to the retrieval focus during AM recollection. Available evidence points to various factors that may influence hemispheric asymmetries in emotion processing that may also affect AMY activity, including emotional valence (negative vs. positive) (Canli et al., 1998), memory processes (encoding vs. retrieval) (Sergerie et al., 2006), and level of processing (automatic vs. elaborated) (Morris et al., 1998; Glascher and Adolphs, 2003; Dyck et al., 2011; Ritchey et al., 2011).

The present AMY lateralization cannot be fully explained by valence effects alone, but could be linked to Glascher’s and Adolphs’ (2003) suggestion that the right AMY is involved in initial, automatic detection of emotions, whereas the left AMY is involved in more elaborated cognitive representation of emotions (see also Morris et al., 1999; Phelps et al., 2001). In the present study, it might be the case that because of its more automatic engagement in emotion detection and processing, and possibly because of different prioritization of processing negative emotions, the right AMY may be less susceptible to modulations of the retrieval focus during recollection of negative AMs. Hence, its response was similarly high regardless of whether the focus was on Emotion or on Context, which was not the case for positive AMs. This interpretation is also consistent with evidence of fast processing of negative stimuli in the AMY (Morris et al., 1999; Vuilleumier et al., 2001).

The absence of significant differences in AMY activity between positive and negative AMs retrieved with an Emotion focus is in line with a valence-independent role of the AMY in the detection and evaluation of relevant and salient emotions (Wager et al., 2008; Lindquist et al., 2012) and with evidence of its involvement in emotional personal recollections (Botzung et al., 2010; Staniloiu and Markowitsch, 2012). Overall, the present data are consistent with both a stronger left AMY engagement for positive and negative AMs, when there is an explicit emphasis on emotional aspects, and a differential right AMY engagement for positive and negative AMs, when emotional processing is not overtly demanded. These findings point to the interplay between emotional valence and retrieval focus, which if not considered together can lead to incomplete conclusions.

(4) Manipulation of the retrieval focus was associated with increased activity for positive and decreased activity for negative AMs in the left HC, in the Emotion condition. Given the evidence linking left hippocampal activity to more detailed recollections (Addis et al., 2004), a possible interpretation is that negative AMs in the Emotion condition are less detailed than the negative AMs in the Context condition. However, this interpretation is not consistent with the present behavioral results, which did not show differences in the scores for Vividness in the Emotion and Context conditions (3.37 and 3.32, respectively). A slightly more nuanced interpretation can be proposed if the effects observed in the HC are considered in the context of evidence regarding its role in processing visual landmarks (Berthoz, 1997) and in binding together contextual and scene-related details (Davachi et al., 2003; Davachi, 2006). Specifically, it could be speculated that greater involvement of the posterior portion of the HC may be solicited to bind contextual details that are detached from emotional aspects (i.e., negative AMs with Context focus). Therefore, the decrease in the left HC for negative memories in the Emotion condition (which had the highest intensity ratings) could be due to an automatic binding of details by arousal (Mather and Sutherland, 2011), which probably required less hippocampal involvement. However, in the case of positive AMs, probably attaining similar level of recollection required increased left hippocampal involvement when the retrieval focus was on Emotion.

#### Caveats

One limitation of the present study is the absence of a Neutral control condition, which limits the interpretation of the findings. This was mainly dictated by the difficulty in identifying enough neutral memories that could be equated with the emotional AMs in terms of their phenomenological properties. The inclusion of neutral memories in future studies could be used as an additional baseline to complement the present findings. Another limitation is that the number of subjects did not allow proper investigation of sex-related differences, which have been addressed in only a handful of AM neuroimaging studies (e.g., Piefke et al., 2005; St Jacques et al., 2011). Future studies should examine whether the effects of retrieval focus and valence are differently affected in women and men.

## Conclusion

In summary, the present study reveals similar and differential involvement of the AMY and HC during the recollection of emotional personal memories, linked to the current retrieval goals and the valence of memories. By shedding light on the role of AMY and HC in these effects, the present findings clarify mixed or inconclusive findings of previous AMs studies in healthy participants, and have the potential to contribute to a better understanding and prevention of affective disorders, which are characterized by an excessive focus on negative AMs.

## Conflict of Interest Statement

The authors declare that the research was conducted in the absence of any commercial or financial relationships that could be construed as a potential conflict of interest.

## Footnotes

^1^Referring to AM studies specifically eliciting the recall of emotional memories, as opposed to “standard” AM studies, referring to studies without explicit instructions to retrieve emotional aspects of memories (see meta-analysis by Svoboda et al., 2006).
